# Pretreatment TACC3 expression in locally advanced rectal cancer decreases the response to neoadjuvant chemoradiotherapy

**DOI:** 10.18632/aging.101585

**Published:** 2018-10-19

**Authors:** Wen-juan Ma, Yang-kui Gu, Jian-hong Peng, Xue-cen Wang, Xin Yue, Zhi-zhong Pan, Gong Chen, Hai-neng Xu, Zhong-guo Zhou, Rong-xin Zhang

**Affiliations:** 1 State Key Laboratory of Oncology in Southern China, Guangzhou, Guangdong, P.R. China; 2 Department of Colorectal Surgery, Sun Yat-sen University Cancer Center, Guangzhou, Guangdong, P.R. China; 3 Collaborative Innovation Center of Cancer Medicine, Guangzhou, Guangdong, P.R. China; 4 Department of Medical Oncology, Sun Yat-sen University Cancer Center, Guangzhou, Guangdong, P.R. China; 5 Department of Hepatobiliary Surgery, Sun Yat-sen University Cancer Center, Guangzhou, Guangdong, P.R. China; 6 Microinvasive Interventional Department, Sun Yat-sen University Cancer Center, Guangzhou, Guangdong, P.R. China; 7 Ovarian Cancer Research Center, Division of Gynecology Oncology, Department of Obstetrics and Gynecology, University of Pennsylvania, Philadelphia, PA19104, USA; * Equal contribution

**Keywords:** rectal cancer, chemoradiotherapy, sensitivity, TACC3, predictor

## Abstract

Chemoradiotherapy combined with surgical resection is the standard treatment for locally advanced rectal cancer, but not all the patients respond to neoadjuvant treatment. Transforming acidic coiled-coil protein-3 (TACC3) is frequently aberrantly expressed in rectal cancer tissue. In this study, we investigated whether TACC3 could serve as a biomarker predictive of the efficacy of chemoradiotherapy. In all, 152 rectal cancer patients with tumor tissue collected at biopsy and set aside before treatment were enrolled in this study. All patients received chemoradiotherapy and surgical resection. Immunohistochemically detected tumoral TACC3 expression significantly decreased sensitivity to chemoradiotherapy [risk ratio (RR) = 2.236, 95% confidence interval (CI): 1.447–3.456; *P =* 0.001] and thus the pathological complete response rate (*P =* 0.001). TACC3 knockdown using specific siRNA enhanced radiotherapy-induced decreases in proliferation and colony formation by HCT116 and SW480 cells and increased the incidence of radiotherapy-induced apoptosis. Cox multivariate analysis showed that TACC3 was a significant prognostic factor for overall survival (*P =* 0.017) and disease-free survival (*P =* 0.020). These findings suggest TACC3 expression may be predictive of chemoradiotherapy sensitivity and prognosis in locally advanced rectal cancer.

## Introduction

Colorectal cancer is one of the most commonly occurring cancers in China [1], with the incidence of rectal cancer being higher than that of colon cancer [2]. Numerous randomized trials, including the CAO/ARO/AIO-04, ACCORD-12, NSABPR-04 and PETACC-6 trials, have been conducted to improve the prognosis of rectal cancer patients, especially those with locally advanced rectal cancer (LARC) [3–5]. The results of these studies showed that chemoradiotherapy (CRT) and total mesorectal excision (TME) significantly reduce local recurrence rates and enable a higher rate of sphincter-saving surgery, thereby improving patients’ quality of life. However, not all patients with LARC can benefit from CRT. According to an earlier study, approximately 30% of LARC patients do not benefit from CRT and even experienced disease progression or metastasis during treatment [6].

How to identify LARC patients most likely to benefit from CRT remains unclear. The selection criteria used by oncologists and other physicians are still based on pelvic magnetic resonance imaging (MRI) and colonoscopic ultrasound findings, such as invasion of all layers of the rectal wall and metastasis to regional lymph nodes and the mesorectal fascia [7]. However, the error rate for pretreatment clinical staging based on MRI or colonoscopic ultrasound is high. More effective, objective markers are therefore needed to identify LARC patients who will or will not benefit from CRT.

Transforming acidic coiled-coil protein-3 (TACC3) is a member of the TACC protein family, which localizes to centrosomes and associates with microtubules [8–10]. The TACC family is essential for interactions between tubulin and microtubules, and proteins in this family are known to play key roles in the regulation of centrosome and microtubule dynamics [11–16]. Three TACC proteins (TACC1-3) have been identified in humans [17–20]. TACC3 reportedly acts as a negative regulator of Notch signaling through binding to CDC10/Ankyrin repeats [8]. High TACC3 expression has been detected in ovarian cancer [21], glioblastoma [22], esophageal squamous cell carcinoma [23], and colorectal cancer [24]. Based on those studies, it appears TACC3 may promote tumor progression by increasing cell proliferation, cancer stem cell populations, and cancer cell migration.

Although the functions of TACC3 in human cancer are unknown, a TACC3-FGFR3 fusion protein has been detected in a subset of glioblastoma multiforme (GBM) [25] and bladder tumor tissues as well as in various cancer cell lines [26]. Other studies have shown that high TACC3 expression enhances the proliferation, migratory/invasive ability and transformation capacity of HeLa cervical cancer cells [27]. In addition, high TACC3 expression is associated with a mesenchymal phenotype, which is typically accompanied by downregulation of the epithelial marker E-cadherin and upregulation of the mesenchymal markers N-cadherin and Vimentin as well as the epithelial-mesenchymal transition (EMT) inducers Snail and Slug [27]. Other studies also demonstrated that TACC3 can be induced by EGF and that EGF-mediated TACC3 induction is dependent on EGFR activation [28]. But although TACC3 expression correlates with poor prognosis, whether TACC3 expression correlates with the response to neoadjuvant chemoradiotherapy remains unknown. In the present study, therefore, we used biopsy samples from rectal cancer patients and colorectal cell lines to assess the relationship between TACC3 expression and CRT sensitivity.

## RESULTS

### General characteristics

**Figure 1:**
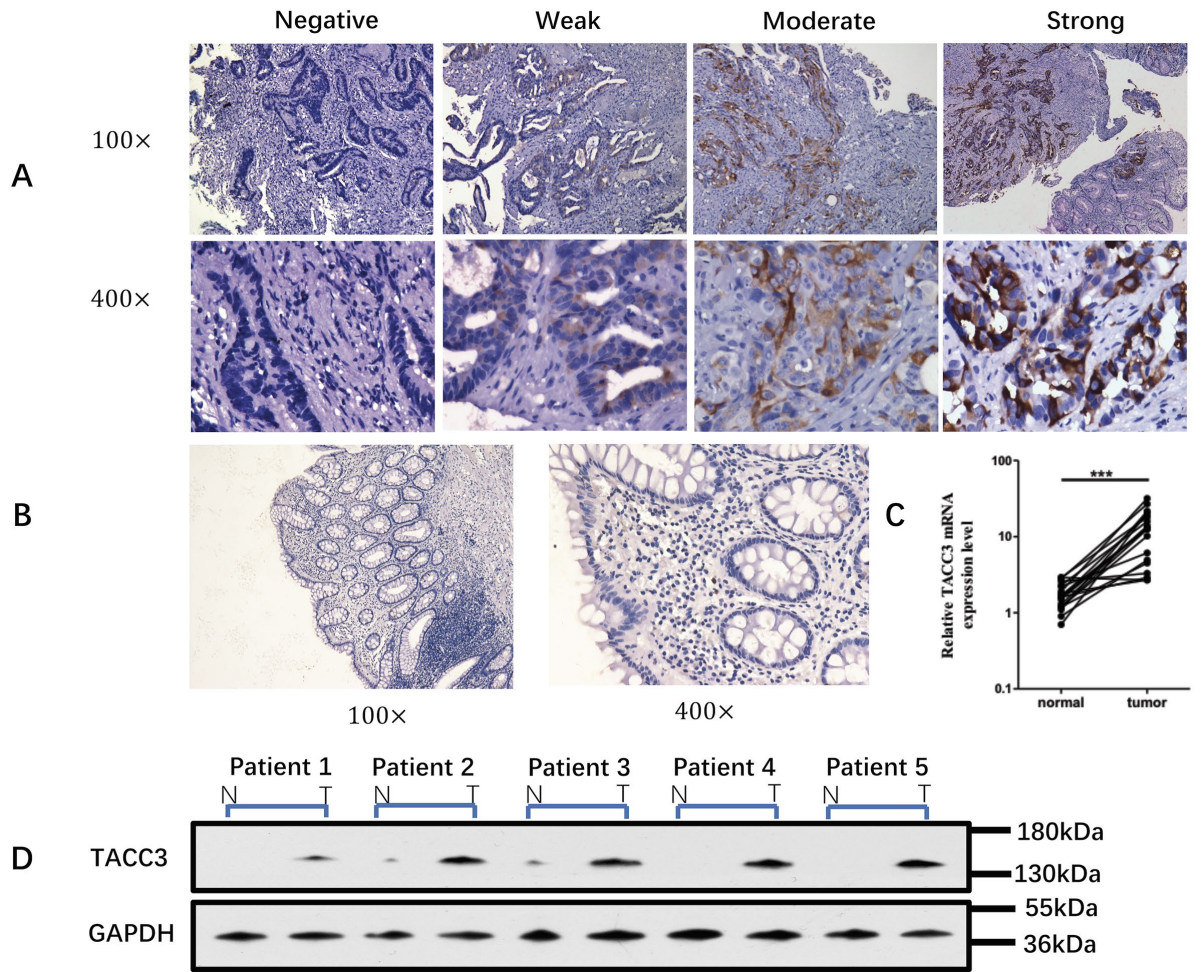
**TACC3 expression in biopsy samples.** (**A**) Expression of TACC3 protein in biopsy tumor tissue and normal tissue from 152 rectal cancer patients before neoadjuvant CRT. The level of TACC3 expression was classified as negative, weak, moderate or strong. (Immunohistochemical staining, 100× and 400×). (**B**) Expression of TACC3 in normal rectal tissue (Immunohistochemical staining, 100× and 400×). (**C**) Levels of TACC3 mRNA in rectal cancer tissue and normal tissue from 20 patients. (**D**) Western blot of biopsy tissue from five rectal cancer patients before neoadjuvant CRT.

**Figure 2:**
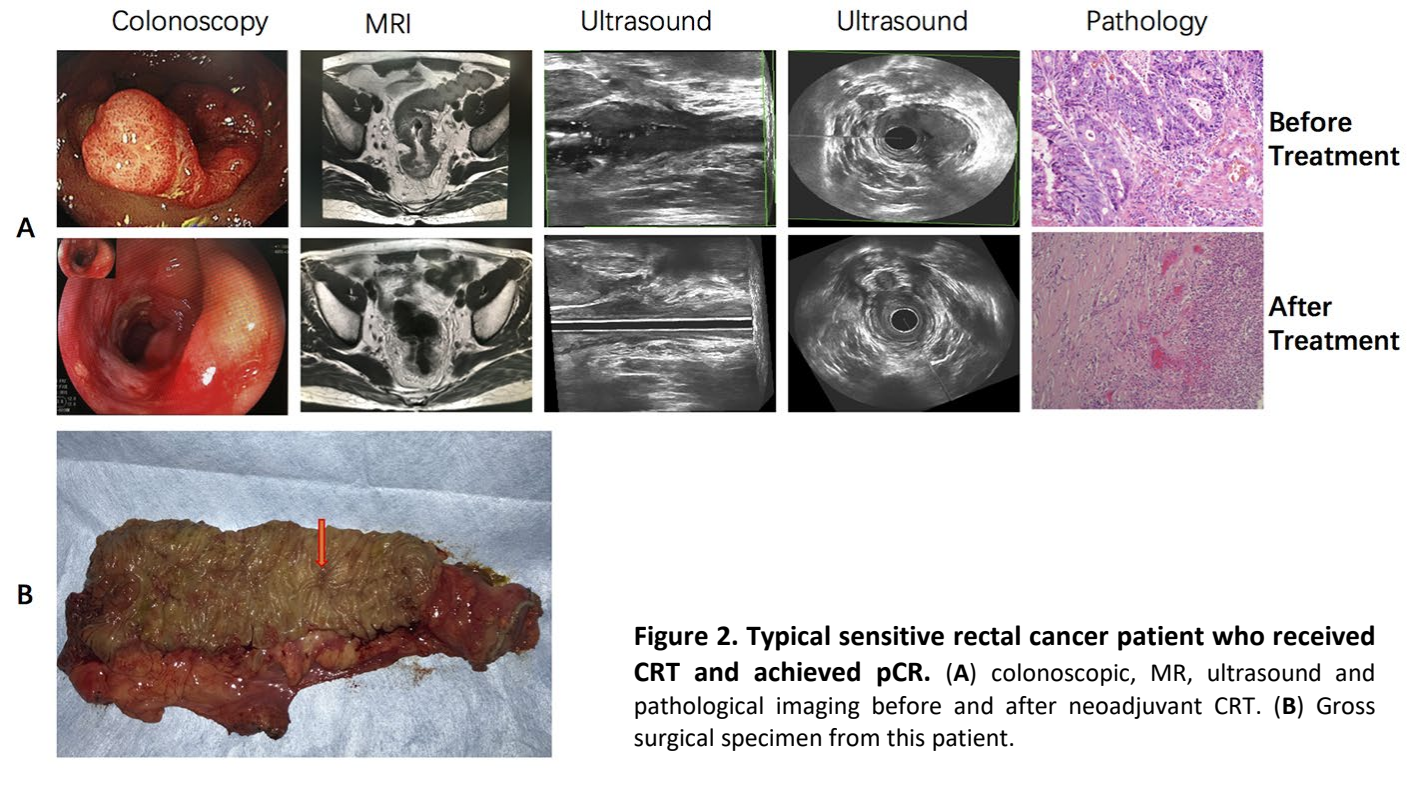
**Typical sensitive rectal cancer patient who received CRT and achieved pCR.** (**A**) colonoscopic, MR, ultrasound and pathological imaging before and after neoadjuvant CRT. (**B**) Gross surgical specimen from this patient.

From May 1, 2003 to May 1, 2016, 152 patients who received neoadjuvant CRT for pathologically confirmed rectal cancer were selected from the Sun Yat-sen University Cancer Center Database. Clinical staging of eligible patients was based on pelvic MRI, ultrasound colonoscopy (with biopsy), CT of the thorax and abdomen, and clinical examination. All examinations were repeated 4 to 5 weeks after CRT for re-staging. Cancer biopsy specimens from 120 patients exhibited TACC3 expression, while those from the remaining 32 were negative for TACC3 expression (Table 1 and Figure 1A). Immunohistochemical and Western blot analyses revealed TACC3 was rarely expressed in normal tissue (Figure 1B). No significant difference in the clinical or pathologic characteristics was detected between the TACC3-positive and TACC3-negative patients, except for the distribution of tumor regression grading (TRG) (*P =* 0.001). Among all patients, 73 were defined as CRT responders, while 79 were defined as CRT non-responders. Forty-one patients achieved a pathological complete response (pCR, 27%). Figure 2 presents representative MR and colonoscopic ultrasound imaging as well as surgical specimens from one patient who achieved a pCR after CRT. Perineural invasion (PNI) (*P =* 0.014) and TACC3 expression (*P* = 0.001) significantly differed between patients who responded to CRT and those who did not. Further details are presented in Table 2.

**Table 1:** Clinical and pathologic characteristics of the TACC3 negative and TACC 3 positive patients.

Characteristic	TACC3 negative (n = 32)				TACC3 positive (n = 120)				P value
	No.	%	Mean	SD	No.	%	Mean	SD	
Sex:									0.286
Male	23	71.9			74	61.7			
Female	9	28.1			46	38.3			
Age			55.78	12.43			54.50	12.73	0.612
T stage*:									0.218
T2	0	0			5	4.2			
T3	23	71.9			60	50.0			
T4b	9	28.1			55	45.8			
N stage*:									0.193
N negative	12	37.5			31	25.8			
N positive	20	62.5			89	74.2			
LN number			7.28	5.21			7.68	5.42	0.740
PNI									0.653
Yes	1	3.1			6	5			
None	31	96.9			114	95			
TD									0.375
Yes	1	3.1			9	7.5			
None	31	96.9			111	92.5			
LVI									0.346
Yes	0	0			7	5.8			
None	32	100			113	94.2			
Pathology types									0.222
Highly differentiated ADC	0	0			1	0.9			
Middle differentiated ADC	29	90.6			100	83.3			
Poorly differentiated ADC	3	9.4			10	8.3			
Undifferentiated ADC	0	0			9	7.5			
TRG									**0.001**
1	28	87.5			13	10.8			
2	2	6.25			30	25.0			
3	2	6.25			63	52.5			
4	0	0			14	11.7			
Survival status:									0.175
Alive	28	87.5			89	74.1			
Dead	4	12.5			31	25.8			
CEA			4.9	5.5			17.5	34.4	0.195
Ca 19-9			19.6	40.1			37.7	98.2	0.320
Neo-chemo regime:									0.866
None	0	0			1	0.8			
Capecitabine	6	18.8			18	15			
CAPOX	24	81.2			95	79.2			
FOLFOX	2	0			5	4.2			
5-FU	0	0			1	0.8			
Neo-chemo cycles:									0.122
0	0	0			1	0.8			
1	0	0			2	1.6			
2	21	65.6			55	45.8			
3	5	15.6			22	18.3			
4	6	18.8			40	33.3			
Adjuvant chemotherapy:									0.951
None	5	15.6			17	14.2			
Capecitabine	2	6.3			15	12.5			
CAPOX	25	78.1			83	69.1			
FOLFOX	0	0			4	3.3			
5-FU	0	0			1	0.9			
Adjuvant chemotherapy cycles:									0.818
0	5	15.6			17	14.2			
1	3	9.4			10	8.3			
2	7	21.9			11	9.2			
3	1	3.1			19	15.8			
4	5	15.6			27	22.5			
5	1	3.1			9	7.5			
6	10	31.3			22	18.3			
8	0	0			4	3.2			
Surgical procedure:									0.993
AR	20	62.5			76	63.3			
APR	11	34.3			40	33.3			
Hartmann	1	3.2			4	3.4			

*The T and N stages were based on MRI before surgery.

TRG = tumor regression grading; 5-FU = 5 fluorouracil; AR = anterior resection; and APR = abdominal perineal resection.

Neo-chemo: Neoadjuvant chemotherapy

ADC: Adenocarcinoma

LN: lymph nodes

PNI: Perineural invasion

LVI: Lymphovascular invasion

**Table 2:** Clinical and pathologic characteristics of the chemoradiotherapy responder and chemoradiotherapy non-responder groups.

Characteristic	Chemoradiotherapy responder group (n = 73)				Chemoradiotherapy non-responder group (n = 79)				P value
	No.	%	Mean	SD	No.	%	Mean	SD	
Sex:									0.814
Male	48	65.8			49	62.0			
Female	25	34.2			30	38.0			
Age			54.71	12.0			54.82	13.2	0.957
T stage*:									0.244
T2	2	2.7			3	3.8			
T3	45	61.6			38	48.1			
T4b	26	35.6			38	53.3			
N stage*:									0.552
N negative	19	26.0			24	30.4			
N positive	54	74.0			55	69.6			
LN number			7.32	5.3			7.85	5.4	
PNI									**0.014**
Yes	73	100			72	91.1			
None	0	0			7	8.9			
TD									0.394
Yes	70	95.9			72	91.1			
None	3	4.1			7	8.9			
LVI									0.143
Yes	84	98.8			84	93.3			
None	1	1.2			6	6.7			
Pathology types of ADC									0.632
Highly differentiated	0	0			1	1.3			
Middle differentiated	62	84.9			67	84.8			
Poorly differentiated	6	8.2			7	13			
Undifferentiated	5	6.8			4	9			
Survival status:									0.755
Alive	57	78.1			60	75.9			
Dead	16	21.9			19	24.1			
CEA			12.3	28.8			17.0	32.8	0.349
Ca 19-9			21.7	35.2			44.6	117.7	0.125
Neoadjuvant chemotherapy regimen									0.957
None	0	0			1	1.3			
Capecitabine	10	13.7			14	17.7			
CAPOX	61	83.6			58	73.4			
FOLFOX	2	2.7			5	6.3			
5-FU	0	0			1	1.3			
Neoadjuvant chemotherapy cycles:									0.022
0	0	0			1	1.3			
1	0	0			2	2.5			
2	29	39.7			47	59.5			
3	17	23.3			10	12.7			
4	27	37.0			19	24.1			
Adjuvant chemotherapy regimen									0.403
None	10	13.7			12	15.2			
Capecitabine	6	8.2			11	13.9			
CAPOX	56	76.7			52	65.9			
FOLFOX	1	1.2			3	3.8			
FOLFIRI	0	0			1	1.3			
Adjuvant chemotherapy cycles:									0.747
0	10	13.7			12	15.4			
1	7	9.6			6	7.7			
2	12	16.4			6	7.7			
3	9	12.3			11	14.1			
4	16	21.9			16	20.5			
5	4	5.5			6	7.7			
6	14	19.2			18	23.1			
8	1	1.4			3	3.8			
Surgical procedure:									0.241
AR	44	60.3			52	65.8			
APR	28	38.4			23	29.1			
Hartmann	1	1.4			4	5.1			
TACC3 expression									**0.001**
Negative	25	34.2			7	8.9			
Weak	28	38.4			32	40.5			
Moderate	16	21.9			33	41.8			
Strong	4	5.5			7	8.9			

*The T and N stages were based on preoperative MRI

TRG, tumor regression grading; 5-FU, 5 fluorouracil; AR, anterior resection; APR, abdominal perineal resection; ADC: adenocarcinoma; LN: lymph node; PNI: perineural invasion; LVI: lymphovascular invasion.

### Association between TACC3 expression and tumor response to CRT

In the logistic regression analysis, there was a significant association between pCR and negative TACC3 expression [risk ratio (RR) = 3.252, 95% confidence interval (CI): 1.918–5.512; *P =* 0.001]. When the response to CRT was analyzed, TACC3 expression [risk ratio (RR) = 2.236, 95% confidence interval (CI): 1.447–3.456; *P =* 0.001] and adjuvant chemotherapy cycles (RR = 0.558, 95% CI: 0.395–0.874; *P =* 0.009) also showed significant association.

### Relationship between TACC3 expression and clinicopathological variables

Analysis of the correlation between TACC3 expression and the clinical and pathological variables revealed no significant correlations between TACC3 expression and clinical T stage (*P* = 0.586), N stage (*P* = 0.059), PNI (*P* = 0.430), the presence of tumor deposits (TDs) (*P* = 0.559), lymphovascular invasion (LVI) (*P* = 0.062), pathological type (*P* = 0.692), carcinoembryonic antigen (CEA) level (*P* = 0.846), CA19-9 level (*P* = 0.629), sex (*P* = 0.983), or age (*P* = 0.792). On the other hand, TACC3 correlated strongly with TRG (*P* = 0.001).

### TACC3 knockdown in HCT116 and SW480 cells and the response to radiotherapy

**Figure 3:**
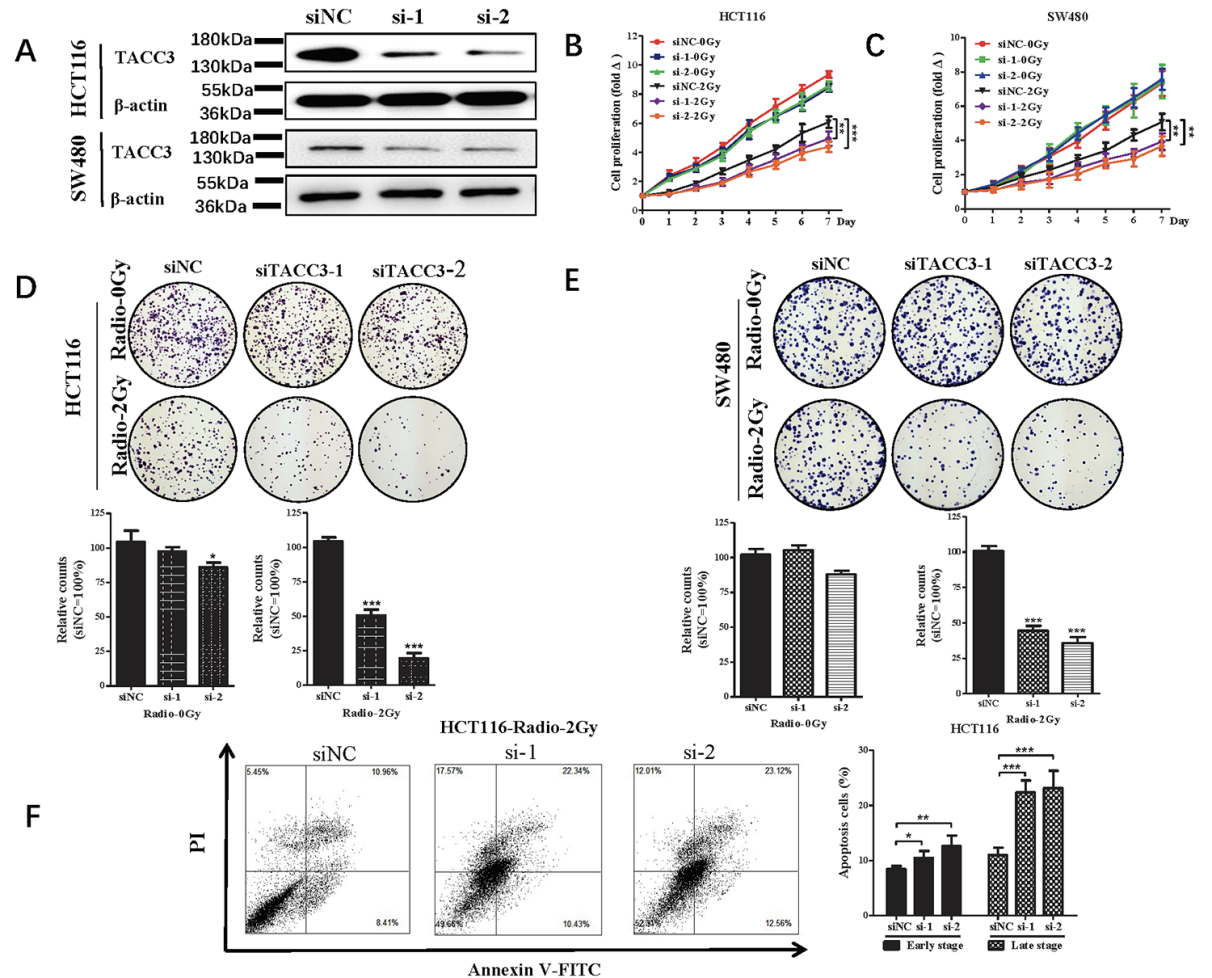
**Effect of radiotherapy on cell proliferation, colony formation and apoptosis after knocking down of TACC3 in HCT116 and SW480 cells.** (**A**) TACC3 protein expression in HCT116 and SW480 knockdown cells. (**B, C**) TACC3 knockdown increases the inhibitory effect of radiotherapy on HCT116 and SW480 cell proliferation measured in CCK-8 assays. (**D, E**) TACC3 Knockdown increases the induced inhibitory effect of radiotherapy on colony formation by HCT116 and SW480 cells. (**F**) TACC3 Knockdown the incidences of radiotherapy-induced early and late apoptosis among HCT116 cells, as measured using flow cytometry with Annexin V/PI double staining. *p<0.05, **p<0.01, ***p<0.001.

To investigate the potential role of TACC3 in determining the sensitivity colorectal cancer (CRC) to radiotherapy, we measured cell proliferation, colony formation and apoptosis after knocking down TACC3 expression using specific siRNA. As shown in Figure 3, transfection of siRNA targeting TACC3 significantly decreased the protein expression of TACC3 in HCT116 and SW480 cells (Figure 3A). TACC3 knockdown significantly increased the inhibitory effect of radiotherapy (Radio-2Gy) on HCT116 and SW480 cell proliferation (Figure 3B and Figure 3C) and on colony formation by the two cell lines (Figure 3D and 3E). TACC3 knockdown also enhanced the apoptotic effect of radiotherapy (Radio-2Gy) on HCT116 (Figure 3F). These results suggest that TACC3 plays a crucial role in mediating radiotherapy resistance and that inhibition of TACC3 could increase the sensitivity of CRC to radiotherapy.

### Correlation between TACC3 expression and overall and disease-free survival

**Figure 4:**
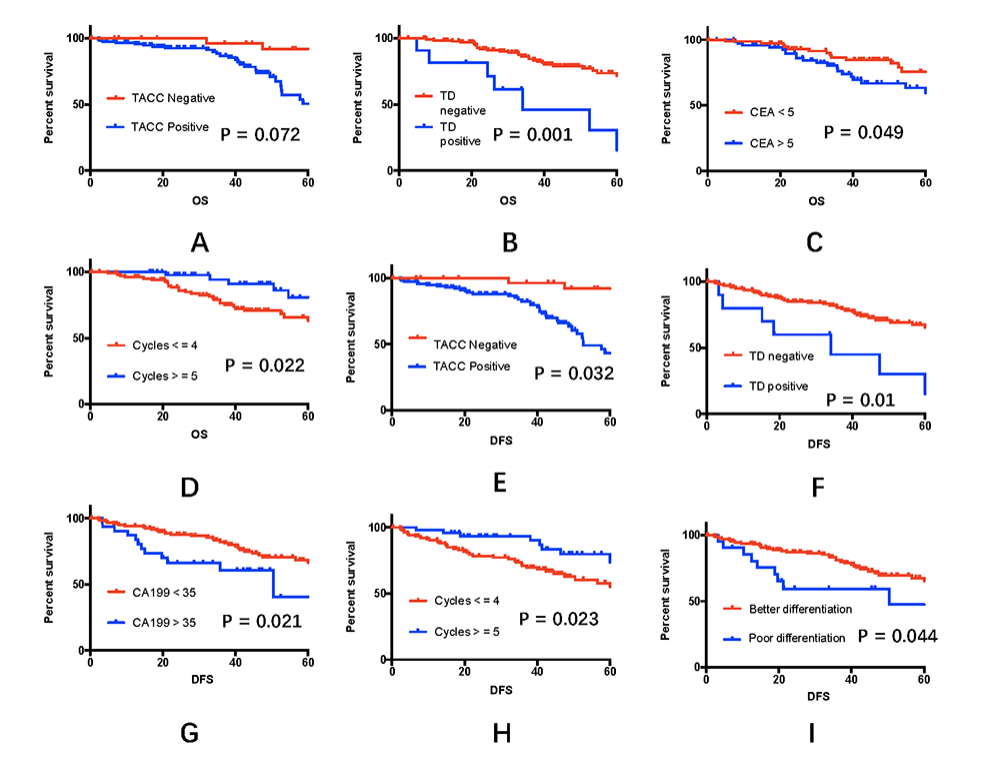
**Effects of clinical variables on 5-year overall and disease-free survival rates.** (**A-D**) Five-year overall survival rates among the 152 rectal cancer patients, taking into account TACC3 expression, the presence of tumor deposits, the CEA level, and adjuvant chemotherapy cycles. (**E-I**) Five-year disease-free survival rates among the 152 rectal cancer patients, taking into account TACC3 expression, the presence of tumor deposits, the CA19-9 level, the number of adjuvant chemotherapy cycles, and tumor differentiation.

All 152 patients were followed-up until November 1, 2017 and were included in the survival analysis. The median follow-up period was 41 months. At the end of the follow-up period, 117 patients were alive, and 35 had died. Univariate analysis to determine possible variables that could affect overall and disease-free survival among rectal cancer patients showed that the CEA level (RR = 1.002, 95%CI: 1.012- 3.831, *P* = 0.049), the presence of TDs (RR = 4.014, 95%CI: 1.712-9.411, *P* = 0.001), and the number of neoadjuvant chemotherapy cycles (RR = 0.828, 95%CI: 0.709-0.967, *P* = 0.022) were strongly related to overall survival. In addition, TACC3 expression (RR = 2.671, 95%CI:1.050-6.793, *P* = 0.032), the CA19-9 level (RR = 1.001,95%CI: 0.998 – 1.003, *P* = 0.021), the presence of TDs (RR = 2.856, 95%CI: 1.242- 6.565, *P* = 0.01), and tumor differentiation (RR = 3.147, 95%CI:0.000-5.814, *P* = 0.044) were strongly related to disease-free survival. Other variables showed no relationship with the survival of rectal cancer patients (additional details are given in Figure 4, the Supplementary Figure S1 and Table 3).

**Table 3:** Univariate and multivariate analyses of prognostic factors for disease-free survival and overall survival in 152 locally advanced rectal cancer patients who underwent chemoradiotherapy as neoadjuvant treatment.

	DFS				OS			
	Univariate		Multivariate		Univariate		Multivariate	
Variable	RR (95% CI)	P	RR (95% CI)	P	RR (95% CI)	P	RR (95% CI)	P
Sex	2.081(1.146-3.778)	0.216			1.495(0.761-2.935)	0.243		
Age	0.978(0.977-1.023)	0.989			1.012(0.985-1.040)	0.384		
T stage	0.799(0.186-3.425)	0.762			0.675(0.151-3.015)	0.959		
N stage	0.715(0.377-1.354)	0.303			1.016(0.489-2.113)	0.966		
LN	1.010(0.960-1.063)	0.704			0.978(0.918-1.042)	0.486		
Positive LN	0.971(0.797-1.183)	0.770			0.888(0.583-1.353)	0.580		
PNI	1.516(0.465-4.946)	0.490			2.016(0.612-6.644)	0.239		
TD	2.856(1.242-6.565)	**0.010**	2.727(1.183-6.286)	0.019	4.014(1.712-9.411)	**0.001**	3.084 (1.323–7.190)	0.017
LVI	1.560(0.482-5.047)	0.458			1.123(0.269-4.698)	0.873		
Tumor Differentiation	3.147(0.000-5.814)	**0.044**			2.908(0.001-2.145)	0.235		
TRG	1.403(0.554-3.553)	0.475			1.318(0.490-3.547)	0.585		
pCR	1.483(0.711-3.094)	0.290			1.198(0.540-2.657)	0.656		
TACC3 expression	2.671(1.050-6.793)	**0.032**	3.140(1.201-8.210)	0.020	2.525(0.888-7.174)	0.072	3.714(1.261-10.93)	0.017
CEA	1.008(1.002-1.015)	0.015	1.008(1.001-1.015)	0.028	1.002(1.012-3.831)	**0.049**		
CA 19-9	1.001(0.998-1.003)	**0.021**			1.001(0.998-1.004)	0.072		
Neo-chemo regime	1.002(0.001-3.180)	0.998			0.348(0.030-3.988)	0.359		
Neo-chemo cycles	0.927(0.649-1.323)	0.675			1.113(0.758-1.636)	0.584		
Adjuvant chemo	1.038(0.400-2.689)	0.939			0.804(0.273-2.367)	0.692		
Adjuvant chemo cycles	0.857(0.750-0.981)	0.023	0.839(0.731-0.962)	0.012	0.828(0.709-0.967)	**0.022**	0.8143(0.696-0.949)	0.009

A Cox multivariate analysis revealed that the significant prognostic factors for overall survival were TACC3 expression (RR = 3.714, 95% CI = 1.261–10.93; *P =* 0.017) and the presence of TDs (RR = 3.084, 95% CI = 1.323–7.190; *P =* 0.017). The CEA level (RR = 1.008, 95% CI = 1.001–1.015; *P =* 0.028), TACC3 expression (RR = 3.140, 95% CI: 1.201–8.210; *P =* 0.020), and the presence of TDs (RR = 2.727, 95% CI = 1.183–6.286; *P* = 0.019) were significant prognostic factors for disease-free survival. Additional details are presented in Table 3.

At the end of the 5-year follow-up period, the overall survival rate for all enrolled patients was 60%, and the disease-free survival rate was 51%. The 5-year overall survival rate was 77% for patients whose tumors were TACC3-negative and 54% for those whose tumors were TACC3-positive (*P* = 0.072, Figure 4A). The 5-year disease-free survival rate was 74% for TACC3-negative patients and 43% for TACC3-positive patients (*P* = 0.032, Figure 4E).

## DISCUSSION

Rectal cancer is considered to be a CRT-sensitive tumor. The standard treatment for LARC is “sandwich” treatment, which consists of neoadjuvant CRT, radical surgical resection and adjuvant chemotherapy. This approach is supported by data that suggest preoperative CRT reduces local recurrence and improves compliance [29]. However, not all LARC patients respond to CRT. There is also a general concern among oncologists about the need to avoid overtreatment of some patients [30]. No reliable biomarker has yet been recommended in the NCCN or ESMO guidelines. It is therefore important to identify biomarkers to help clinicians select patients who will respond to the current standard treatment.

TACC3, which is expressed specifically in colorectal cancer tissue, is reported to have a strong relationship with worse clinical stage, T classification and M classification [24]. In the present study, clinical T and N stages showed no relationship with TACC3 expression (*P* = 0.586 and 0.059 for T stage and N stage, respectively). The most likely explanation for this finding is the high error rate in clinical staging. Our findings are consistent with earlier studies in that TACC3 was specifically expressed in colorectal cancer tissue [24]. Approximately 21.05% of rectal cancer patients did not express TACC3, while 7.24% of rectal cancer patients exhibited strong expression of TACC3.

Whether TACC3 expression has a relationship with CRT sensitivity has not been previously studied. Our results indicate that rectal cancer patients overexpressing TACC3 were more likely to experience CRT resistance [risk ratio (RR) = 2.236, 95% confidence interval (CI): 1.447–3.456; *P =* 0.001]. A possible mechanism for this phenomenon may be TACC3-mediated activation of the p38-p53-p21 stress signaling pathway [31]. Consistent with that idea, reports suggest p53 status is related to the effect of CRT in both rectal [32] and anal [33] cancer. The p53 status could be a determinant of radiotherapy sensitivity during G1-phase of the cell cycle, as suppression of TACC3 induces G1 arrest. Another explanation is that TACC3 overexpression promotes a decrease in E-cadherin expression and increases in expression of Snail and Slug, which play critical roles in EMT during embryonic development. Consistent with that idea, TACC3 is associated with EMT in both osteogenic sarcoma and cervical cancer [27]. Moreover, EMT is reported to play a key role in CRT sensitivity in rectal cancer [34].

We observed that TACC3 knockdown significantly increased the incidence of early stage apoptosis induced by radiotherapy, and that the increase in late stage apoptosis was even more remarkable. We speculate that TACC3 may be involved in double-strand DNA break repair and in protecting the integrity of the cellular shape and structure. TACC3 knockdown rendered CRC cells sensitive to radiotherapy, increased cell membrane permeability, and accelerated the apoptosis process. In that context, a non-viral delivery system, such as nanoparticles, could be a new and safe way to transfer a TACC3 inhibitor or siRNA to tumor cells in combination with traditional CRT [35,36]. Cationic polymers can be used to load DNA or siRNA into positively charged nanoparticles. For example, DMMA (2,3-Dimethylmaleicanhydride)-amidized polymer was used as a shell to encapsulate positively charged PEI-siRNA complexes. At pH 6.8, the charge reversal of the DMMA-amidized polymer from negative to positive enabled discharge of positively charged PEI-siRNA complexes, which were then endocytosed into cancer cells. leading to gene silencing and reduced protein expression. The inorganic nanoparticles possess many characteristics suitable for therapeutic gene or siRNA delivery, including high control lability, good biocompatibility, multifunctionality, and high surface area/volume ratio. However, nanoparticles continue to have important limitations. For example, there is substantial variation in transfection efficiency, depending on the experimental parameters and the investigator. So, there is still a long way to go.

Our results indicate that higher TACC3 expression is strongly related to poorer disease-free survival (*P* = 0.032) and that rectal cancer patients not expressing TACC3 tend to have better overall survival, though the difference is not significance (*P* = 0.072). TACC3 may thus be a prognostic factor, but additional studies with larger patient populations and longer follow-up times are required. Other prognostic factors for survival include the CEA level (*P* = 0.049), the presence of TDs (*P* = 0.001) and the number of adjuvant chemotherapy cycles (*P* = 0.022). We did not detect a relationship between TRG and survival. Many still argue that TRG could be a strong prognostic factor for LARC patients. One previous study showed that the American Joint Committee on Cancer (AJCC) TRG system is an appropriate tumor regression grading system that predicts local recurrence in LARC patients who undergo chemoradiotherapy [37]. In other studies, however, no correlation between TRG and survival was detected [38–40].

Its retrospective nature is a key limitation of this study. The number of enrolled patients was also limited, as it is very difficult to set aside biopsy tissue from all rectal cancer patients. The mechanism by which TACC3 suppresses CRT sensitivity and the associated signaling pathways are still under investigation.

## Conclusion

In summary, our findings suggest TACC3 has the potential to serve as a biomarker of CRT sensitivity in LARC patients and should be explored as a potential therapeutic target in cases of CRT resistance.

## MATERIALS AND METHODS

### Patients and treatment

From May 28, 2003 to May 31, 2016, all rectal cancer patients who received neoadjuvant CRT were screened from the Sun Yat-sen University Cancer Center Database. The inclusion criteria were: histologically confirmed rectal cancer; no detected distant metastasis; T3 or T4b diagnosed by MRI, ultrasound or colonoscopy, with or without positive regional lymph nodes; no severe primary tumor-related symptoms (severe bleeding, bowel obstruction or perforation); resectable primary tumor; Eastern Cooperative Oncology Group performance status of 0 or 1; age between 18 and 75 years; and written informed consent. The exclusion criteria were: high rectal cancer (tumor >10 cm from the anal verge; tumor-related symptoms (bleeding requiring transfusion, bowel obstruction, or tumor perforation); patients not eligible for surgery or CRT; distant metastasis; history of another primary cancer (not including skin or cervical cancer); inability to complete standard neoadjuvant CRT; death within one month after surgery; did not undergo radical surgical resection; or lost to follow-up within 6 months. Prior to neoadjuvant treatment, tumor tissue collected during colonoscopy was deposited in the tissue bank at Sun Yat-sen University Cancer Center. Adjuvant chemotherapy was recommended for all patients, including those who achieved pCR.

All patients received standard radiotherapy. A total dose of 50 Gy was delivered to the gross tumor volume, and 46 Gy were delivered to the clinical target volume. High-energy photons (6 or 8 MV) were delivered in 25 daily fractions, from Monday to Friday, over a period of 5 weeks. Chemotherapy was recommended to all patients receiving radiotherapy.

The neoadjuvant chemotherapy regimens consisted of capecitabine, CapOX (capecitabine and oxaliplatin), FOLFOX, and 5-fluorouracil (5-FU). Surgical resection entailed anterior resection, abdominal perineal resection, or the Hartmann procedure. Both open surgery and laparoscopic surgery were acceptable.

**Figure 5:**
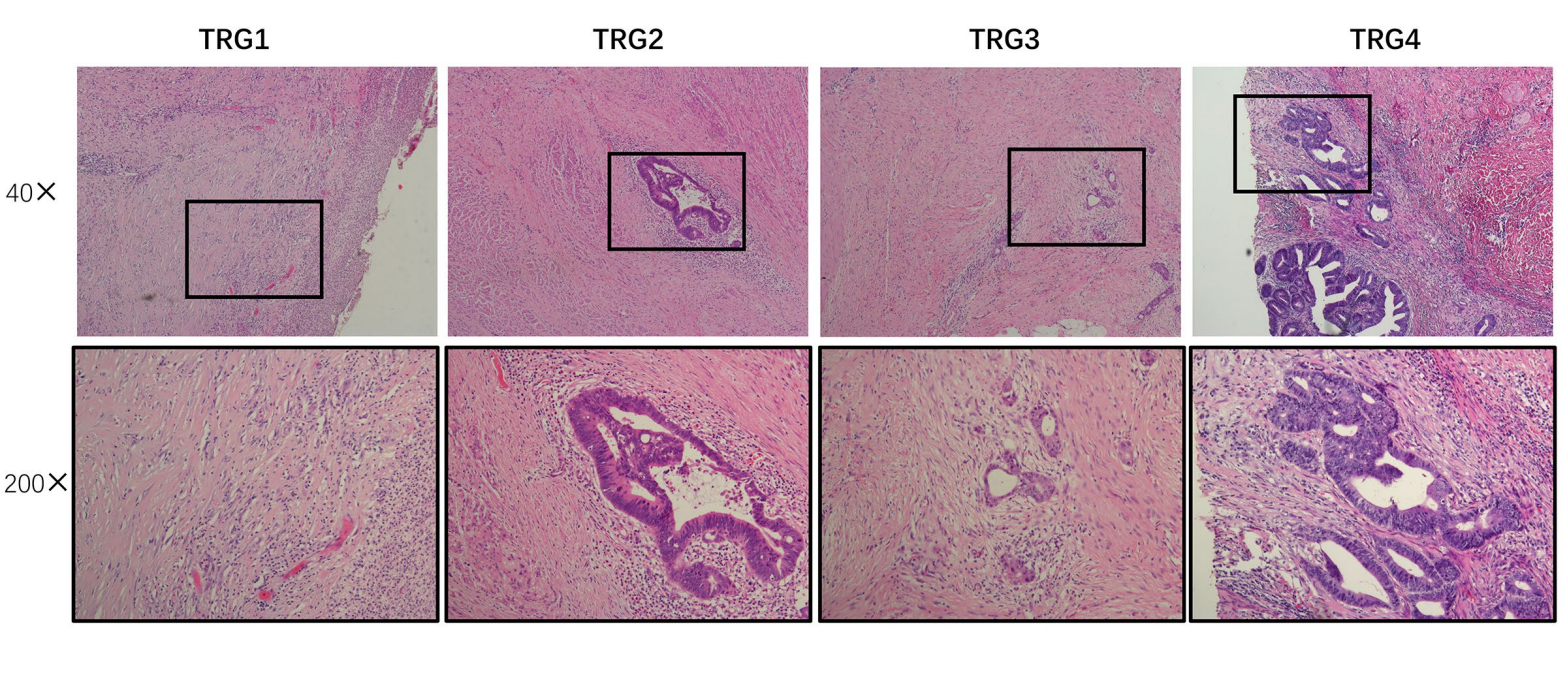
**Assessment of tumor regression.** Using AJCC tumor regression grading (TRG), tumors were classified into four histological tumor regression grades based on the amount of vital tumor tissue and the ratio of fibrosis as follows: TRG 0, complete regression and absence of viable cancer cells; TRG 1, presence of only small clusters or single cancer cells; TRG 2, presence of residual cancer cells but with predominant fibrosis; and TRG 3, minimal or no decrease in tumor cells or extensive residual cancer.

This study used the AJCC TRG system to evaluate patient response to CRT. Tumor regression was classified into four histologic TRGs based on vital tumor tissue and the ratio of fibrosis after CRT: TRG 0, complete regression and the absence of viable cancer cells; TRG 1, the presence of only small clusters or single cancer cells; TRG 2, the presence of residual cancer cells with predominant fibrosis; and TRG 3, minimal or no decrease in the tumor cells or extensive residual cancer (Figure 5). Patients classified as TRG 0 or 1 were considered chemotherapy responders, while those classified as TRG 2 or 3 were chemotherapy non-responders.

### Follow-up

All patients were followed-up in accordance with NCCN guidelines. During the first two years, all patients were followed-up at every 3 months. Thereafter, patients were followed-up every 6 months for the next 3 years and annually after 5 years. During each visit, the patients underwent a physical examination, testing for CEA and CA19-9 levels, and abdominal and pelvic ultrasound examinations. Colonoscopy after resection was recommended at approximately one year, or at 3 months if it was not performed preoperatively due to the presence of an obstructing lesion. A repeat colonoscopy was typically recommended at 3 years and every 5 years thereafter, unless follow-up colonoscopy indicated the presence of an advanced adenoma in which case the colonoscopy was repeated in one year. All patients underwent chest, abdominal, and pelvic CT scans annually until 5 years after surgery.

### Immunohistochemical analysis

Immunohistochemistry was performed as previously reported [24]. Paraffin-embedded specimens were serially cut into three 4-μm-thick sections. One section was used for routine hematoxylin and eosin staining, while the other two sections were used for staining using the streptavidin peroxidase (SP) immunohistochemistry method. The experimental procedure was performed according to the manufacturer’s instructions for each reagent kit. After the specimens were deparaffinized and rehydrated, they were washed three times in PBS and boiled in a high-pressure cooker for 2.5 min in EDTA buffer (pH 8.0) for antigen retrieval. Non-specific binding was then blocked using 5% BSA, after which the sections were incubated consecutively with the primary antibody, secondary antibody, and enzyme-labeled SP. Finally, the sections were developed using 3,3’-diaminobenzidine (DAB) and counterstained with hematoxylin. The stained sections were cleared, mounted, and examined under a microscope.

The primary antibody solution consisted of a rabbit anti-human TACC3 monoclonal antibody (1:400 dilution; ab134154, Abcam) in blocking buffer, which was incubated with the sections at 4°C overnight in a moist chamber. Blocking buffer without the primary antibody was used as a negative control.

Each slide was evaluated using the immunohistochemistry scoring system used in our previous study [41]. If the conclusions of the two pathologists differed, a third pathologist evaluated each case independently and decided the final score. Based on the staining intensity, the results were classified as negative, weak, moderate, or strong. Negative was defined as no TACC3 expression, while weak, moderate and strong encompassed positive TACC3 expression.

### Western blotting

Western blotting was conducted as previously described [24]. Briefly, equal amounts of protein were separated by 8% SDS-PAGE. The resolved proteins were electrophoretically transferred onto 0.22-μm PVDF membranes, after which the membranes were incubated with rabbit anti-human TACC3 (1:1000; Sigma), anti-β-actin (1:1000; CST) and anti-GAPDH (1:3000; Thermo Fisher Scientific Inc) antibodies.

### Quantitative real-time RT PCR

Total RNA was isolated from tissues using the TRIzol reagent (Invitrogen, Carlsbad, CA, USA). cDNA was obtained by reverse transcription using the M-MLV Kit (Promega, Madison, WI, USA) for RT-qPCR. SYBR Green (Bio-Rad) was used for RT-PCR gene expression analysis, carried out on Bio-Rad CFXplatform. Relative mRNA levels were normalized to GAPDH mRNA. The primer sequences are listed below.

TACC3:

S: CCTCTTCAAGCGTTTTGAGAAAC,

AS: GCCCTCCTGGGTGATCCTT;

GAPDH:

S: GAAGGTGAAGGTCGGAGTC,

AS: GAAGATGGTGATGGGATTTC.

### Transfection of siRNAs

Cells in 6-well plates were transfected with 100 nM specific or nontarget control siRNA using Lipofectamine 2000 according to the manufacturer’s protocol. The transfected cells were incubated for 24 h at 37°C in FBS-free medium. The target sequences of the TACC3 siRNAs were CCACAGATCTGAACTCCAT for siTACC3-1 (si1) and GGATTACCTGGAGCAGTTT for siTACC3-2 (si2). The efficiency of siRNA interference was assessed by immunoblotting using an anti-TACC3 antibody.

### Colony formation assay

Cells were seeded into 6-well plates (500-1000 cells per well) and cultured for 7-14 days. After fixation in methanol for 10 min, the colonies formed were stained with 0.5% crystal violet in 20% methanol and counted. All the experiments were performed independently in triplicate.

### CCK-8

Suspension of transfected cells was dispensed into 96-well plates (1,000 cells/well, 100 μL) and then incubated for 1 to 7 days (humidified atmosphere, 37°C, 5% CO2). Thereafter, 10 μL of CCK8 (Dojindo Laboratories, Kumamoto, Japan) was added to each well, and the cells were incubated for an additional 4 h at 37°C. The absorbance at 460 nm (A460) was then assayed using a scanning multiwell spectrophotometer (Thermo Scientific).

### Apoptosis assay

An Annexin V/PI apoptosis kit was used to assess the incidence of apoptotic cell death. Cells incubated for 24 h in 6-well plates (5 x 10^4^ cells/well) were exposed to radiation and then incubated for an additional 24 h. Approximately 1 x 10^5^ cells were stained for 5 min with Annexin V/PI in the dark and then analyzed using a two-color flow cytometric assay. The Annexin V/PI apoptosis kit detects the externalization of phosphatidylserine in apoptotic cells using recombinant FITC-conjugated annexin V (green) and dead cells using red-fluorescent propidium iodide (PI; red). After treatment with the two probes, early stage apoptotic cells fluoresced green, while late stage apoptotic cells red fluoresced red and green. Live cells showed little or no fluorescence [42]. The data were analyzed using the Cell Quest program (Beckman Coulter).

### Statistical analysis

The clinical and follow-up data were analyzed using SPSS v19.0. The χ^2^, continuity correction χ^2^, and Fisher’s exact tests were used to assess the patients’ baseline variables. The significance of the variables was tested using multivariate Cox regression and logistic regression models. Overall survival was defined as the interval between surgical resection and death or the end of follow-up. Disease-free survival was defined as the interval between surgical resection and recurrence, metastasis, or the end of follow-up. Values of P < 0.05 were considered significant.

### Ethics approval and consent to participate

This study was approved by the Institutional Research Ethics Committee of Sun Yat-sen University Cancer Center. Informed consent to use tissue samples collected before the initial treatment were obtained from all patients.

### Availability of data and materials

The datasets analyzed during the current study were available from the corresponding author on reasonable request. Anyone who is interested in the information should contact zhangrx@sysucc.org.cn or zhouzhg@sysucc.org.cn.

## SUPPLEMENTARY MATERIAL

Supplementary Figure S1

## References

[1] Chen W, Zheng R, Baade PD, Zhang S, Zeng H, Bray F, Jemal A, Yu XQ, He J. Cancer statistics in China, 2015. CA Cancer J Clin. 2016; 66:115–32. 10.3322/caac.21338 26808342

[2] Tao K, Yang J, Guo Z, Hu Y, Sheng H, Gao H, Yu H. Prognostic value of miR-221-3p, miR-342-3p and miR-491-5p expression in colon cancer. Am J Transl Res. 2014; 6:391–401. 25075256 PMC4113501

[3] Rödel C, Liersch T, Becker H, Fietkau R, Hohenberger W, Hothorn T, Graeven U, Arnold D, Lang-Welzenbach M, Raab HR, Sülberg H, Wittekind C, Potapov S, et al, and German Rectal Cancer Study Group. Preoperative chemoradiotherapy and postoperative chemotherapy with fluorouracil and oxaliplatin versus fluorouracil alone in locally advanced rectal cancer: initial results of the German CAO/ARO/AIO-04 randomised phase 3 trial. Lancet Oncol. 2012; 13:679–87. 10.1016/S1470-2045(12)70187-0 22627104

[4] Gérard JP, Azria D, Gourgou-Bourgade S, Martel-Laffay I, Hennequin C, Etienne PL, Vendrely V, François E, de La Roche G, Bouché O, Mirabel X, Denis B, Mineur L, et al. Comparison of two neoadjuvant chemoradiotherapy regimens for locally advanced rectal cancer: results of the phase III trial ACCORD 12/0405-Prodige 2. J Clin Oncol. 2010; 28:1638–44. 10.1200/JCO.2009.25.8376 20194850

[5] Roh M, Yothers G, O’Connell M, Beart R, Pitot H, Shields A, Parda D, Sharif S, Allegra C, Petrelli N, Landry JC, Ryan DP, Arora A, et al. The impact of capecitabine and oxaliplatin in the preoperative multimodality treatment in patients with carcinoma of the rectum: NSABP R-04. J Clin Oncol. 2011; 29:3503. 10.1200/jco.2011.29.15_suppl.350321844503

[6] Yu Z, Zhang C, Wang H, Xing J, Gong H, Yu E, Zhang W, Zhang X, Cao G, Fu C. Multidrug resistance-associated protein 3 confers resistance to chemoradiotherapy for rectal cancer by regulating reactive oxygen species and caspase-3-dependent apoptotic pathway. Cancer Lett. 2014; 353:182–93. 10.1016/j.canlet.2014.07.025 25088576

[7] Glimelius B, Tiret E, Cervantes A, Arnold D, and ESMO Guidelines Working Group. Rectal cancer: ESMO Clinical Practice Guidelines for diagnosis, treatment and follow-up. Ann Oncol. 2013 (Suppl 6); 24:vi81–88. 10.1093/annonc/mdt240 24078665

[8] Bargo S, Raafat A, McCurdy D, Amirjazil I, Shu Y, Traicoff J, Plant J, Vonderhaar BK, Callahan R. Transforming acidic coiled-coil protein-3 (Tacc3) acts as a negative regulator of Notch signaling through binding to CDC10/Ankyrin repeats. Biochem Biophys Res Commun. 2010; 400:606–12. 10.1016/j.bbrc.2010.08.111 20804727 PMC2964058

[9] McKeveney PJ, Hodges VM, Mullan RN, Maxwell P, Simpson D, Thompson A, Winter PC, Lappin TR, Maxwell AP. Characterization and localization of expression of an erythropoietin-induced gene, ERIC-1/TACC3, identified in erythroid precursor cells. Br J Haematol. 2001; 112:1016–24. 10.1046/j.1365-2141.2001.02644.x 11298601

[10] LeRoy PJ, Hunter JJ, Hoar KM, Burke KE, Shinde V, Ruan J, Bowman D, Galvin K, Ecsedy JA. Localization of human TACC3 to mitotic spindles is mediated by phosphorylation on Ser558 by Aurora A: a novel pharmacodynamic method for measuring Aurora A activity. Cancer Res. 2007; 67:5362–70. 10.1158/0008-5472.CAN-07-0122 17545617

[11] Gergely F, Karlsson C, Still I, Cowell J, Kilmartin J, Raff JW. The TACC domain identifies a family of centrosomal proteins that can interact with microtubules. Proc Natl Acad Sci USA. 2000; 97:14352–57. 10.1073/pnas.97.26.14352 11121038 PMC18922

[12] Lee MJ, Gergely F, Jeffers K, Peak-Chew SY, Raff JW. Msps/XMAP215 interacts with the centrosomal protein D-TACC to regulate microtubule behaviour. Nat Cell Biol. 2001; 3:643–49. 10.1038/35083033 11433296

[13] Sadek CM, Pelto-Huikko M, Tujague M, Steffensen KR, Wennerholm M, Gustafsson JÅ. TACC3 expression is tightly regulated during early differentiation. Gene Expr Patterns. 2003; 3:203–11. 10.1016/S1567-133X(02)00066-2 12711550

[14] Schneider L, Essmann F, Kletke A, Rio P, Hanenberg H, Wetzel W, Schulze-Osthoff K, Nürnberg B, Piekorz RP. The transforming acidic coiled coil 3 protein is essential for spindle-dependent chromosome alignment and mitotic survival. J Biol Chem. 2007; 282:29273–83. 10.1074/jbc.M704151200 17675670

[15] Gergely F, Draviam VM, Raff JW. The ch-TOG/XMAP215 protein is essential for spindle pole organization in human somatic cells. Genes Dev. 2003; 17:336–41. 10.1101/gad.245603 12569123 PMC195983

[16] Yao R, Natsume Y, Noda T. TACC3 is required for the proper mitosis of sclerotome mesenchymal cells during formation of the axial skeleton. Cancer Sci. 2007; 98:555–62. 10.1111/j.1349-7006.2007.00433.x 17359303 PMC11158658

[17] Ha GH, Kim JL, Breuer EK. Transforming acidic coiled-coil proteins (TACCs) in human cancer. Cancer Lett. 2013; 336:24–33. 10.1016/j.canlet.2013.04.022 23624299

[18] Still IH, Hamilton M, Vince P, Wolfman A, Cowell JK. Cloning of TACC1, an embryonically expressed, potentially transforming coiled coil containing gene, from the 8p11 breast cancer amplicon. Oncogene. 1999; 18:4032–38. 10.1038/sj.onc.1202801 10435627

[19] Lauffart B, Gangisetty O, Still IH. Molecular cloning, genomic structure and interactions of the putative breast tumor suppressor TACC2. Genomics. 2003; 81:192–201. 10.1016/S0888-7543(02)00039-3 12620397

[20] Still IH, Vince P, Cowell JK. The third member of the transforming acidic coiled coil-containing gene family, TACC3, maps in 4p16, close to translocation breakpoints in multiple myeloma, and is upregulated in various cancer cell lines. Genomics. 1999; 58:165–70. 10.1006/geno.1999.5829 10366448

[21] Lauffart B, Vaughan MM, Eddy R, Chervinsky D, DiCioccio RA, Black JD, Still IH. Aberrations of TACC1 and TACC3 are associated with ovarian cancer. BMC Womens Health. 2005; 5:8. 10.1186/1472-6874-5-8 15918899 PMC1175095

[22] Duncan CG, Killela PJ, Payne CA, Lampson B, Chen WC, Liu J, Solomon D, Waldman T, Towers AJ, Gregory SG, McDonald KL, McLendon RE, Bigner DD, Yan H. Integrated genomic analyses identify ERRFI1 and TACC3 as glioblastoma-targeted genes. Oncotarget. 2010; 1:265–77. 10.18632/oncotarget.137 21113414 PMC2992381

[23] Huang ZL, Lin ZR, Xiao YR, Cao X, Zhu LC, Zeng MS, Zhong Q, Wen ZS. High expression of TACC3 in esophageal squamous cell carcinoma correlates with poor prognosis. Oncotarget. 2015; 6:6850–61. 10.18632/oncotarget.3190 25760075 PMC4466654

[24] Du Y, Liu L, Wang C, Kuang B, Yan S, Zhou A, Wen C, Chen J, Wu Y, Yang X, Feng G, Liu B, Iwamoto A, et al. TACC3 promotes colorectal cancer tumourigenesis and correlates with poor prognosis. Oncotarget. 2016; 7:41885–97. 10.18632/oncotarget.9628 27248823 PMC5173103

[25] Singh D, Chan JM, Zoppoli P, Niola F, Sullivan R, Castano A, Liu EM, Reichel J, Porrati P, Pellegatta S, Qiu K, Gao Z, Ceccarelli M, et al. Transforming fusions of FGFR and TACC genes in human glioblastoma. Science. 2012; 337:1231–35. 10.1126/science.1220834 22837387 PMC3677224

[26] Williams SV, Hurst CD, Knowles MA. Oncogenic FGFR3 gene fusions in bladder cancer. Hum Mol Genet. 2013; 22:795–803. 10.1093/hmg/dds486 23175443 PMC3554204

[27] Ha GH, Park JS, Breuer EK. TACC3 promotes epithelial-mesenchymal transition (EMT) through the activation of PI3K/Akt and ERK signaling pathways. Cancer Lett. 2013; 332:63–73. 10.1016/j.canlet.2013.01.013 23348690

[28] Ha GH, Kim JL, Breuer EK. TACC3 is essential for EGF-mediated EMT in cervical cancer. PLoS One. 2013; 8:e70353. 10.1371/journal.pone.0070353 23936413 PMC3731346

[29] Sauer R, Liersch T, Merkel S, Fietkau R, Hohenberger W, Hess C, Becker H, Raab HR, Villanueva MT, Witzigmann H, Wittekind C, Beissbarth T, Rödel C. Preoperative versus postoperative chemoradiotherapy for locally advanced rectal cancer: results of the German CAO/ARO/AIO-94 randomized phase III trial after a median follow-up of 11 years. J Clin Oncol. 2012; 30:1926–33. 10.1200/JCO.2011.40.1836 22529255

[30] Ryan JE, Warrier SK, Lynch AC, Heriot AG. Assessing pathological complete response to neoadjuvant chemoradiotherapy in locally advanced rectal cancer: a systematic review. Colorectal Dis. 2015; 17:849–61. 10.1111/codi.13081 26260213

[31] Suhail TV, Singh P, Manna TK. Suppression of centrosome protein TACC3 induces G1 arrest and cell death through activation of p38-p53-p21 stress signaling pathway. Eur J Cell Biol. 2015; 94:90–100. 10.1016/j.ejcb.2014.12.001 25613365

[32] Chow OS, Kuk D, Keskin M, Smith JJ, Camacho N, Pelossof R, Chen CT, Chen Z, Avila K, Weiser MR, Berger MF, Patil S, Bergsland E, Garcia-Aguilar J. KRAS and combined KRAS/TP53 mutations in locally advanced rectal cancer are independently associated with decreased response to neoadjuvant therapy. Ann Surg Oncol. 2016; 23:2548–55. 10.1245/s10434-016-5205-4 27020587 PMC5047012

[33] Gilbert DC, Williams A, Allan K, Stokoe J, Jackson T, Linsdall S, Bailey CM, Summers J. p16INK4A, p53, EGFR expression and KRAS mutation status in squamous cell cancers of the anus: correlation with outcomes following chemo-radiotherapy. Radiother Oncol. 2013; 109:146–51. 10.1016/j.radonc.2013.08.002 24021343

[34] Bhangu A, Wood G, Brown G, Darzi A, Tekkis P, Goldin R. The role of epithelial mesenchymal transition and resistance to neoadjuvant therapy in locally advanced rectal cancer. Colorectal Dis. 2014; 16:O133–43. 10.1111/codi.12482 24617665

[35] Wang K, Huang Q, Qiu F, Sui M. Non-viral delivery systems for the application in p53 cancer gene therapy. Curr Med Chem. 2015; 22:4118–36. 10.2174/0929867322666151001121601 26423086

[36] Zhang B, Wang K, Si J, Sui M, Shen Y. (2014). Charge-reversal polymers for biodelivery: Wiley-VCH Verlag GmbH & Co. KGaA).

[37] Trakarnsanga A, Gönen M, Shia J, Nash GM, Temple LK, Guillem JG, Paty PB, Goodman KA, Wu A, Gollub M, Segal N, Saltz L, Garcia-Aguilar J, Weiser MR. Comparison of tumor regression grade systems for locally advanced rectal cancer after multimodality treatment. J Natl Cancer Inst. 2014; 106:106. 10.1093/jnci/dju248 25249540 PMC4271114

[38] Abdul-Jalil KI, Sheehan KM, Kehoe J, Cummins R, O’Grady A, McNamara DA, Deasy J, Breathnach O, Grogan L, O’Neill BD, Faul C, Parker I, Kay EW, et al. The prognostic value of tumour regression grade following neoadjuvant chemoradiation therapy for rectal cancer. Colorectal Dis. 2014; 16:O16–25. 10.1111/codi.12439 24119076

[39] Swellengrebel HA, Bosch SL, Cats A, Vincent AD, Dewit LG, Verwaal VJ, Nagtegaal ID, Marijnen CA. Tumour regression grading after chemoradiotherapy for locally advanced rectal cancer: a near pathologic complete response does not translate into good clinical outcome. Radiother Oncol. 2014; 112:44–51. 10.1016/j.radonc.2014.05.010 25018000

[40] Appelt AL, Vogelius IR, Pløen J, Rafaelsen SR, Lindebjerg J, Havelund BM, Bentzen SM, Jakobsen A. Long-term results of a randomized trial in locally advanced rectal cancer: no benefit from adding a brachytherapy boost. Int J Radiat Oncol Biol Phys. 2014; 90:110–18. 10.1016/j.ijrobp.2014.05.023 25015203 PMC4159435

[41] Peng JH, Fang YJ, Li CX, Ou QJ, Jiang W, Lu SX, Lu ZH, Li PX, Yun JP, Zhang RX, Pan ZZ, Wan S. A scoring system based on artificial neural network for predicting 10-year survival in stage II A colon cancer patients after radical surgery. Oncotarget. 2016; 7:22939–47. 10.18632/oncotarget.8217 27008710 PMC5008413

[42] Lecoeur H, Prévost MC, Gougeon ML. Oncosis is associated with exposure of phosphatidylserine residues on the outside layer of the plasma membrane: a reconsideration of the specificity of the annexin V/propidium iodide assay. Cytometry. 2001; 44:65–72. 10.1002/1097-0320(20010501)44:1<65::AID-CYTO1083>3.0.CO;2-Q 11309810

