# Supplementary material for: Pretreatment TACC3 expression in locally advanced rectal cancer decreases the response to neoadjuvant chemoradiotherapy
Source: Aging (Albany NY). 2018 Oct 19;10(10):2755–71. doi: 10.18632/aging.101585 (PMC6224241; doi:10.18632/aging.101585)
Supplement: Supplementary Figure S1 [file aging-10-101585-s001.pdf]

SUPPLEMENTARY MATERIAL

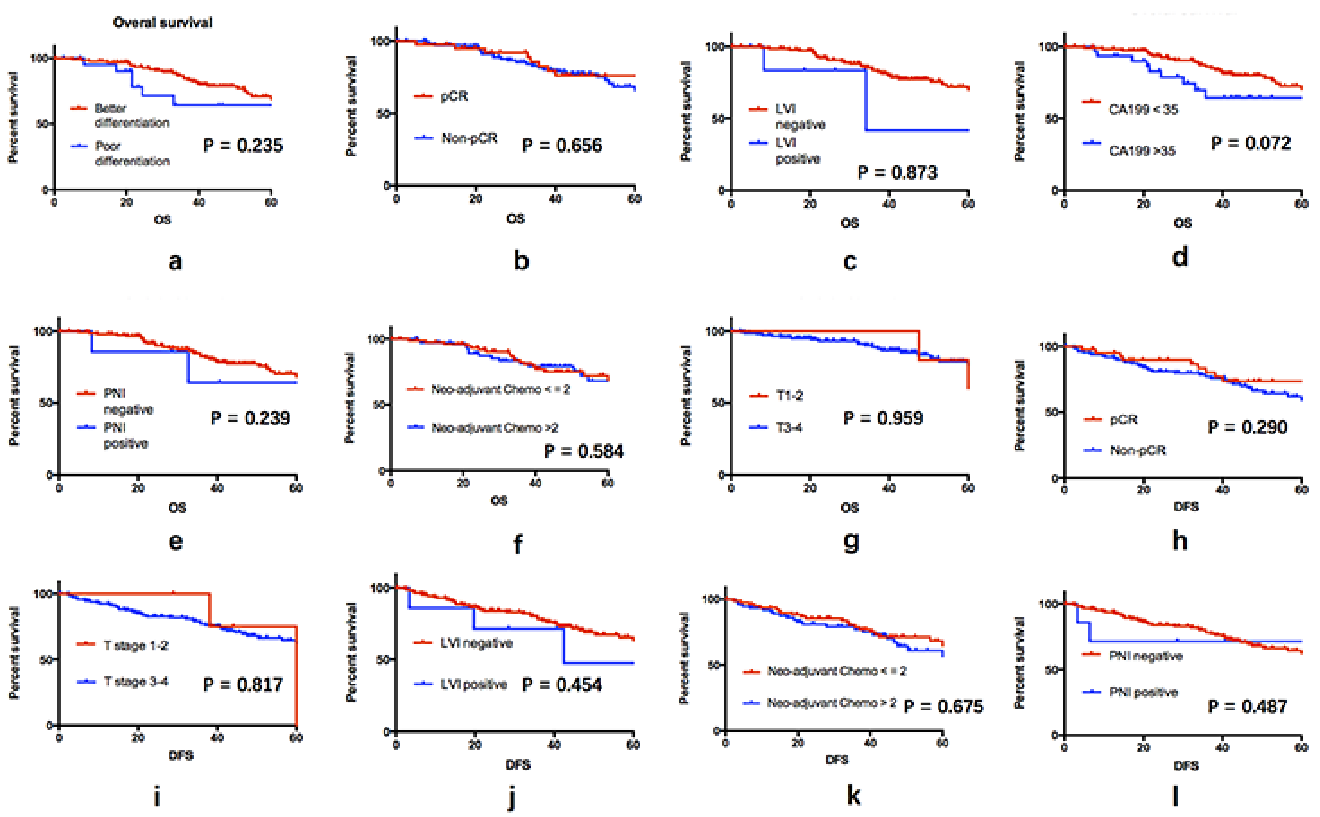

Supplementary Figure S1. Effects of clinical variables not shown in Figure 4 on 5-year overall and disease-free survival rates. (**A-D**) Five-year overall survival rate among the 152 rectal cancer patients, taking into account tumor differentiation, pCR status, LVI status, and the CA19-9 level. (**E-L**) Five-year disease-free survival rate among the 152 rectal cancer patients, taking into account PNI status, neoadjuvant chemotherapy cycles, T stage, pCR status, T stage, LVI status, number of neoadjuvant chemotherapy cycles, and PNI status.
